# Thalidomide Attenuates Mast Cell Activation by Upregulating SHP-1 Signaling and Interfering with the Action of CRBN

**DOI:** 10.3390/cells12030469

**Published:** 2023-02-01

**Authors:** Hyeun-Wook Chang, Kyeong-Hwa Sim, Youn-Ju Lee

**Affiliations:** 1 College of Pharmacy, Yeungnam University, Gyeongsan 38541, Republic of Korea; 2 Department of Pharmacology, School of Medicine, Daegu Catholic University, Daegu 42472, Republic of Korea

**Keywords:** thalidomide, mast cell, allergy, Src homology 2 domain-containing phosphatase-1, adenosine monophosphate-activated protein kinase, cereblon

## Abstract

Allergy is a chronic inflammatory disease, and its incidence has increased worldwide in recent years. Thalidomide, which was initially used as an anti-emetic drug but was withdrawn due to its teratogenic effects, is now used to treat blood cancers. Although the anti-inflammatory and immunomodulatory properties of thalidomide have been reported, little is known about its influence on the mast cell-mediated allergic reaction. In the present study, we aimed to evaluate the anti-allergic activity of thalidomide and the underlying mechanism using mouse bone marrow-derived mast cells (BMMCs) and passive cutaneous anaphylaxis (PCA) mouse models. Thalidomide markedly decreased the degranulation and release of lipid mediators and cytokines in IgE/Ag-stimulated BMMCs, with concurrent inhibition of FcεRI-mediated positive signaling pathways including Syk and activation of negative signaling pathways including AMP-activated protein kinase (AMPK) and SH2 tyrosine phosphatase-1 (SHP-1). The knockdown of AMPK or SHP-1 with specific siRNA diminished the inhibitory effects of thalidomide on BMMC activation. By contrast, the knockdown of cereblon (CRBN), which is the primary target protein of thalidomide, augmented the effects of thalidomide. Thalidomide reduced the interactions of CRBN with Syk and AMPK promoted by FcεRI crosslinking, thereby relieving the suppression of AMPK signaling and suppressing Syk signaling. Furthermore, oral thalidomide treatment suppressed the PCA reaction in mice. In conclusion, thalidomide suppresses FcεRI-mediated mast cell activation by activating the AMPK and SHP-1 pathways and antagonizing the action of CRBN, indicating that it is a potential anti-allergic agent.

## 1. Introduction

Mast cells are well-recognized initiators of IgE-triggered allergic inflammation and anaphylactic responses via their release of a broad array of mediators, including preformed and granule-stored mediators, newly-synthesized eicosanoids, and pro-inflammatory cytokines, upon binding of antigen (Ag) to Ag-specific IgE bound to the high-affinity IgE receptor (FcεRI) [1,2].

FcεRI-mediated mast cell activation is regulated by coordinated signaling pathways. Ag-induced crosslinking of IgE-bound FcεRI activates FcεRI-associated tyrosine kinases such as Lyn and Fyn, which subsequently recruit and activate Syk. Then, Syk phosphorylates linker for activation of T cells (LAT), resulting in assembly and sequential activation of a downstream signaling complex [3,4]. These signaling pathways are counter-regulated by negative signaling pathways including those involving SH2 tyrosine phosphatases (SHPs), inhibitory kinases such as Csk, and ubiquitin ligases [5,6]. In addition, we previously reported that AMP-activated protein kinase (AMPK) negatively modulates FcεRI initiated signaling pathways in mast cells [7,8]. In resting cells, AMPK and liver kinase B1 (LKB1), an upstream kinase of AMPK, halt mast cell activation by associating with sirtuin 1 (Sirt1), forming a trimeric complex [9]. In IgE/Ag-stimulated cells, the activation of AMPK and LKB1 is downregulated by Fyn and ERK. Upregulation of the LKB1/AMPK pathway ameliorates mast cell-mediated allergic responses by downregulating PLCγ1, ERK1/2, JNK, and IKK activities [7,8,9].

The kinase activity of AMPK is regulated by phosphorylation of the α subunit and allosteric changes in the β and γ subunits [10,11,12]. In addition, AMPK activity is negatively regulated by a direct interaction with cereblon (CRBN), a substrate receptor of the Cullin 4-RING (CRL4) E3 ubiquitin ligase complex [13,14]. CRBN disrupts the assembly of AMPK subunits by directly binding to AMPKα both in vitro and in vivo [15,16]. The biological functions of CRBN are complex and diverse. CRBN deficiency elevates pro-inflammatory gene expression in macrophages and monocytes [17,18], decreases the survival of mice in response to Toll-like receptor 4 stimulation [17], and ameliorates ethanol-increased expression of pro-inflammatory genes in the liver [16], indicating that CRBN has pro- and anti-inflammatory roles depending on the pathological condition. CRBN is the primary target of thalidomide [14,19].

Thalidomide was initially used as an anti-emetic drug for pregnant women, although it was withdrawn from the pharmaceutical market due to its serious teratogenic effects [20,21,22]. However, a growing body of research provides evidence that thalidomide possesses immunomodulatory and anti-oncogenic properties [23,24,25]. Recent studies showed that thalidomide elicits therapeutic effects in various inflammatory disease models including allergic asthma [26,27] and rosacea-like skin inflammation [28]. In addition, a multicenter phase II study reported that thalidomide improves mast cell mediator-related symptoms in systemic mastocytosis, which is characterized by the abnormal accumulation of mast cells [29]. Moreover, thalidomide attenuates high glucose-induced renal damage and extracellular matrix protein synthesis by activating the AMPK pathway [30,31]. However, the effect of thalidomide on mast cell activation and the underlying mechanisms have not been clearly established.

In the present study, we examined the effects of thalidomide on FcεRI-mediated allergic responses and its possible mechanisms of action, focusing on AMPK, CRBN, and SHP-1 in bone marrow-derived mast cells (BMMCs) and passive cutaneous anaphylaxis (PCA) animal models.

## 2. Materials and Methods

### 2.1. Reagents

Thalidomide (#T144), dinitrophenyl human serum albumin (DNP-HSA, #A6661), DNP-specific monoclonal IgE (#D8406), and p-nitrophenly-N-acetyl-β-D-glucosaminide (#N9376) were obtained from Sigma-Aldrich (St. Louis, MO, USA) and NSC-87877 (#565851) was obtained from Calbiochem (San Diego, CA, USA). Thalidomide was prepared by dissolving in dimethyl sulfoxide (DMSO) and the final concentration of DMSO was adjusted to 0.1% (*v/v*) in culture medium. DMSO was used as a vehicle control in all cases.

### 2.2. Mice

Seven-week-old male Balb/cJ and ICR mice were obtained from Daehan Biolink Co., Ltd. (Eumseong-gun, Chungbuk, Republic of Korea). After one week of adaptation, animal experiments were performed in accordance with protocols approved by the Institutional Animal Care and Use Committee of the Daegu Catholic University Medical Center.

### 2.3. Preparation and Activation of Mouse Bone Marrow-Derived Mast Cells (BMMCs) and Rat Basophilic-like Cells (RBL2H3)

BMMCs were prepared from Balb/cJ as previously described [7]. Cells were isolated from femur bones of mice with RPMI 1640 medium and cultured in RPMI 1640 medium containing 10% FBS, 1% antibiotic/antifungal agent, 100 μM MEM non-essential amino acid solution, 10 mM HEPES, and 20% PWM-SCM (pokeweed mitogen-spleen cell conditioned medium) as a source of IL-3 at 37 °C with 5% CO_2_ for 4 weeks. RBL2H3 cells were cultured in DMEM supplemented with 10% FBS and 1% antibiotic/antifungal agent. For cell stimulation, cells were sensitized with 500 ng/mL of mouse anti-DNP IgE for 2 h and then stimulated with 100 ng/mL of DNP-HSA for different times. Thalidomide was added 1 h prior to the stimulation of cells with DNP-HSA.

### 2.4. β-Hexosaminidase Assay

The activity of β-hexosaminidase was measured to determine the level of degranulation. After stimulation of IgE-sensitized BMMCs (2 × 10^6^ cells/mL) with DNP-HSA for 15 min, the culture medium was collected by centrifugation at 1200× *g*. The same number of control BMMCs were lysed by repeated freezing/thawing and used to measure total levels of degranulation. Twenty-five μL of supernatants were incubated with 50 μL *p*-nitrophenyl-N-acetyl-β-D-glucosaminide (1.3 mg/mL) in 0.1 M citric acid buffer (pH 4.5) at 37 °C for 1 h. The enzyme reaction was stopped by adding 0.2 M glycine buffer (pH 10.7). The absorbance was measured at 405 nm on a Molecular Devices spectramax iD5 (San Jose, CA, USA). Data were expressed as a percentage of total values.

### 2.5. Determination of Eicosanoids and Cytokines

IgE-sensitized BMMCs (1 × 10^6^ cells/mL) were stimulated with DNP-HSA as indicated above and, then, culture media were collected. The levels of released leukotriene C_4_ (LTC_4_), prostaglandin D_2_ (PGD_2_), tumor necrosis factor α (TNF-α), and interleukin-6 (IL-6) were assessed using ELISA kits according to the manufactures’ instruction. ELISA kits for LTC_4_ (#501070) and PGD_2_ (#512011) were obtained from Cayman Chemicals (Ann Arbor, MI, USA) and TNF-α (MTA00B) and IL-6 (M6000B) were obtained from R&D Systems (Minneapolis, MN, USA).

### 2.6. Estimation of Intracellular Ca^2+^ Level

Calcium mobilization was determined using the Calcium Assay Kit (Enzo Life Sciences, Ann Arbor, MI, USA, #ENZ-51017) according to the manufacturer’s instruction [32]. IgE-sensitized BMMCs were washed twice with PBS and were preincubated with FluoForte Dye or dye-loading solution for 1 h. After washing with HBSS, BMMCs (5 × 10^4^ cells/well) were seeded into 96-well microplates and stimulated with DNP-HSA for 5 min. The fluorescence was measured at 485/520 nm on a Molecular Devices spectramax iD5 (San Jose, CA, USA).

### 2.7. Immunoblotting (IB)

BMMCs were washed with ice-cold PBS and incubated with lysis buffer (20 mM HEPES, pH 7.5, 1 mM EGTA, 1 mM EDTA, 10 mM NaF, 2 mM MgCl_2_, 150 mM NaCl, 10 mM KCl, 1 mM Na_3_VO_4_, 1 mM DTT, 1 mM benzamide, 1 mM PMSF, 10 μg/mL aprotinin, 10 μg/mL leupeptin, 10 mM β-glycerophosphate, and 1% (*v*/*v*) NP-40) on ice for 15 min with vortexing every 5 min. Cell lysates were obtained by centrifugation at 15,000× *g* for 15 min and protein concentration was measured by detergent-compatible protein assay (Bio-Rad, Hercules, CA, USA, #5000112) Denatured proteins using 4X Laemmli sample buffer (Bio-Rad, Hercules, CA, USA, #1610747) were separated on 10% SDS-PAGE and were transferred to a nitrocellulose membrane (0.45 μm) using a semi-dry transfer cell (Bio-Rad). After blocking with 5% non-fat dry milk in 1x TBST (10 mM Tris-HCl, pH 7.5, 150 mM NaCl, 0.05% Tween-20) at room temperature for 1 h, membranes were horizontally cut to probe proteins with different molecular weights. Membranes were incubated with primary antibodies at 4 °C overnight and then incubated with horseradish peroxidase-conjugated secondary antibody for 1 h at room temperature. The band intensities were analyzed by the Chemi-Doc XRS imaging system (Bio-Rad, Hercules, CA, USA). The membranes were stripped and reprobed with anti-GAPDH antibody as needed. The results are expressed as arbitrary units after normalization to GAPDH levels, which was used as loading control for each gel. The primary antibodies used were as follows: Antibodies against p-Akt (#4058), Akt (#9272), p-ACC (#3661), ACC (#3662), p-AMPKα (#2531), AMPKα (#2532), p-LKB1 (#3482), LKB1 (#3050), p-ERK (#4337), ERK (#9102), p-IKK (#2697), p-JNK (#9251), JNK (#9258), p-p38 MAPK (#9215), p38 MAPK (#9212), p-PLCγ1 (#2821), and GAPDH (#2118) were purchased from Cell Signaling Technology (Danvers, MA). Antibodies against Fyn (#sc-434), IgG (#sc-2025, 2027), IKK (#sc-7607), LAT (#sc-53550), Lyn (#sc-376100), PLCγ1 (#sc-7290), SHP-1 (#sc-7289), Syk (#sc-929, sc-1240), and p-tyrosine (pY) (#sc-7020) were purchased from Santa Cruz Biotechnology (Santa Cruz, CA); anti-CRBN antibody was from Thermo Fisher scientific (Pittsburgh, PA, USA, #PA5-106287). The secondary antibodies, goat anti-rabbit IgG antibody (#GTX213110-0), and goat anti-mouse IgG antibody (#GTX213111-01) were purchased from Gene Tex (Irvine, CA, USA).

### 2.8. Immunoprecipitation (IP)

Cell lysates obtained using lysis buffer were preincubated with protein G/A agarose (Calbiochem, San Diego, CA, USA, #IP05) for 2 h at 4 °C. Agarose beads were spin-downed and supernatant was collected for immunoprecipitation. Corresponding antibodies (4 μL) were added to 100 μL of cell lysate (1 μg/μL) and were incubated with gentle agitation at 4 °C overnight. The immunocomplexes were captured by adding 25 μL of protein G/A agarose for 2 h at 4 °C. The mixtures were washed with washing buffer (150 mM Sodium chloride, 1% Triton X-100, 1% sodium deoxycholate, 0.1% SDS, 50 mM Tris-HCl, pH 7.5, and 2 mM EDTA) three times and then resuspended in lysis buffer. The immunocomplexes were subjected to immunoblotting, as described above, as needed. Antibodies used for IP were as follows: Antibodies against AMPKα (#sc-74461), Syk (#sc-929, sc-1240), LAT (#sc-7948), Fyn (#sc-28791), Lyn (#sc-28790), and SHP-1 (#sc-287) were from Santa Cruz Biotechnology (Santa Cruz, CA, USA).

### 2.9. Transfection of siRNA

Genetic knockdown was performed using mouse-specific siRNAs (Santa Cruz Biotechnology, Santa Cruz, CA, USA) and a non-specific siRNA (ThermoFisher, Santa Cruz Biotechnology, Santa Cruz, CA, USA). IgE-sensitized BMMCs were cultured in serum-free medium for 16 h and transfected with 100 nM of AMPKα (50 nM α1(#sc-29674) and 50 nM α2 (#sc-38924)), CRBN (#sc-142562), and SHP-1 (#sc-29479) siRNAs or a non-specific siRNA (Mock) (#12935300), as a negative control using DharmaFECT3 Transfection reagent (Dharmacon, Lafayette, CO, #T-2003-03). After 48 h, cells were stimulated with DNP-HSA for indicated times [32].

### 2.10. IgE/Ag-Mediated Passive Cutaneous Anaphylaxis (PCA) in Mice

To evaluate the effect of thalidomide on IgE/Ag-mediated type I hypersensitivity reaction in vivo, PCA reaction was induced as described previously [7]. ICR mice were intradermally injected in the ear with 80 ng of mouse anti-DNP IgE. After 24 h, IgE-sensitized mice were orally administered two different doses (10 and 25 mg/kg) of thalidomide or 50 mg/kg of fexofenadine (Korea Pharma, Seoul, Republic of Korea), followed 1 h later by being intravenously challenged with 60 μg of DNP-HSA containing evans blue (1% in PBS) (*n* = 6 per group). Thalidomide and fexofenadine were prepared in 0.5% of carboxymethyl cellulose. Control group was administered an equivalent volume of a vehicle (0.5% of carboxymethyl cellulose). After anesthetization of mice, blood was collected and the levels of serum LTC_4_ and PGD_2_ were analyzed as described above. To determine dye extravasation, ears were removed and incubated with 400 μL of formamide at 63 °C overnight, and the absorbance of extracted dye was measured at 630 nm on a Molecular Devices spectramax iD5 (San Jose, CA, USA).

### 2.11. Statistical Analysis

Statistical significance was determined by unpaired Student’s *t* test (two-tailed) for comparisons between two groups and one-way ANOVA with Tukey’s post hoc test for multiple comparisons using GraphPad Prism 4.0 [32]. *p* < 0.05 was considered statistically significant.

## 3. Results

### 3.1. Thalidomide Inhibits IgE/Ag-Stimulated Mast Cell and Basophil Activation

**Figure 1:**
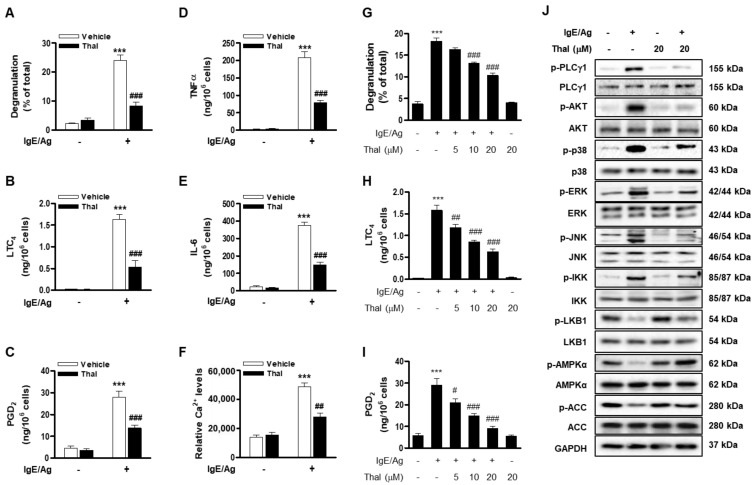
Thalidomide attenuates FcεRI-mediated mast cell activation. BMMCs were sensitized with anti-DNP IgE and treated with 20 μM of thalidomide (Thal) (**A**–**F**,**J**) or different concentrations of thalidomide (**G**–**I**) for 1 h followed by stimulation with DNP-HSA (Ag). Degranulation (**A**,**G**) and secretion of LTC_4_ (**B**,**H**), PGD_2_ (**C**,**I**), TNFα (**D**), and IL-6 (**E**), and the level of intracellular Ca^2+^ (**F**), were evaluated. Cell lysates were subjected to immunoblotting with the indicated antibodies (**J**). Data (**A**–**I**) represent the means ± SEM from at least three independent experiments with different BMMCs (*** p < 0.01 vs. control; ^#^ *p* < 0.05, ^##^ *p* < 0.01 and ^###^ *p* < 0.001 vs. IgE/Ag alone), and the immunoblots (**J**) are representative of three independent experiments.

To examine the effect of thalidomide on FcεRI-mediated BMMC activation, IgE-sensitized BMMCs were stimulated with Ag (DNP-HSA) in the presence or absence of 20 μM thalidomide. While thalidomide alone had no significant effect on mast cell activation, thalidomide significantly diminished IgE/Ag-stimulated degranulation as well as the production of LTC_4_, PGD_2_, TNF-α, and IL-6, and the intracellular Ca^2+^ level (Figure 1A–F). The inhibitory effects of thalidomide on degranulation and eicosanoid production were concentration-dependent (Figure 1G–I). Thalidomide did not cause cytotoxicity up to 20 μM (Figure S1). Concurrently, thalidomide dramatically downregulated IgE/Ag-activated FcεRI downstream signaling molecules including PLCγ1, AKT, p38, ERK1/2, JNK, and IKK (Figure 1G and Figure S2). In addition, thalidomide substantially enhanced the IgE/Ag-suppressed phosphorylation of LKB1, AMPK, and acetyl-CoA carboxylase (ACC), a downstream target of AMPK. Our previous studies reported that LKB1/AMPK do not affect AKT and p38 in BMMCs [7,8]. Therefore, these results suggest that thalidomide suppresses FcεRI-mediated BMMC activation via both AMPK-dependent and -independent pathways. Moreover, thalidomide attenuated IgE/Ag-stimulated degranulation and eicosanoid production in RBL2H3 cells (Figure S3), suggesting that thalidomide attenuates the IgE/Ag-stimulated activation of mast cells and basophils.

### 3.2. The Inhibitory Effects of Thalidomide on IgE/Ag-Stimulated Mast Cell Activation Are Partly Mediated by the AMPK Pathway

**Figure 2:**
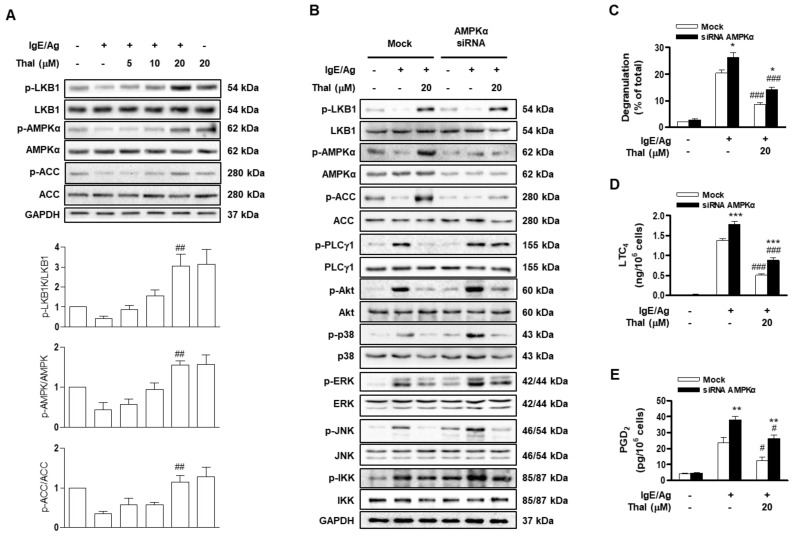
AMPKα knockdown decreases the inhibitory effects of thalidomide on IgE/Ag-stimulated mast cell activation. BMMCs were sensitized with anti-DNP IgE and treated with various concentrations (5, 10, and 20 μM) of thalidomide (Thal) for 1 h (**A**) or IgE-sensitized BMMCs were treated with AMPKα siRNA or non-specific (Mock) siRNA for 48 h and then treated with 20 μM of Thal (**B**–**E**) followed by stimulation with DNP-HSA (Ag) for 15 min. The levels of phosphorylated or total protein were determined by immunoblotting (**A**,**B**). The representative blots from three independent experiments were presented. The bar graph presents the ratio of band intensity of phosphorylated signaling molecules to those of the total proteins (**A**). Degranulation (**C**) and secretion of LTC_4_ (**D**) and PGD_2_ (**E**) were evaluated as described in the Materials and Methods section. Data represent the means ± SEM (n = 3, * *p* < 0.05, ** *p* < 0.01, and *** *p* < 0.001 vs. Mock in each treatment; ^#^ *p* < 0.05, ^##^ *p* < 0.01, and ^###^ *p* < 0.001 vs. IgE/Ag alone in each group).

To confirm the effect of thalidomide on the AMPK pathway, IgE-sensitized BMMCs were pretreated with or without various concentrations of thalidomide (5–20 μM) for 1 h prior to stimulation with Ag. Thalidomide increased the phosphorylation of LKB1, AMPK, and ACC in a concentration-dependent manner (Figure 2A). The involvement of AMPK in the inhibitory effects of thalidomide on BMMC activation was examined by using AMPK siRNA. As expected, AMPKα siRNA substantially decreased the phosphorylation levels of AMPK and ACC, as well as the AMPK protein level, without affecting the phosphorylation level of LKB1. AMPKα siRNA partially reversed the inhibitory effects of thalidomide on the FcεRI downstream signaling molecules PLCγ1, ERK1/2, JNK, and IKK (Figure 2B and Figure S4). Accordingly, IgE/Ag-induced degranulation and generation of LTC_4_ and PGD_2_ were significantly higher in AMPKα siRNA-treated BMMCs than in mock-treated BMMCs (Figure 2C–E). The inhibitory effects of thalidomide on BMMC activation were largely, although not completely, abolished by AMPKα siRNA (Figure 2C–E). These results suggest that thalidomide inhibits FcεRI-mediated BMMC activation partly by regulating AMPK-dependent pathways.

### 3.3. Thalidomide Relieves Suppression of the AMPK Pathway by Interfering with the Association of AMPK with CRBN in IgE/Ag-Stimulated BMMCs

**Figure 3:**
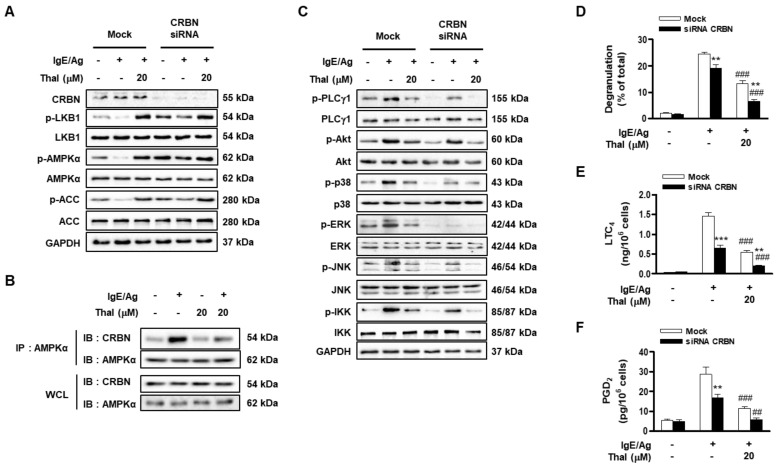
CRBN knockdown enhances the inhibitory effects of thalidomide on IgE/Ag-stimulated mast cell activation. IgE-sensitized BMMCs were transfected with CRBN siRNA or non-specific (Mock) siRNA for 48 h. Then, BMMCs were stimulated with DNP-HSA (Ag) for 15 min in the presence or absence of thalidomide (Thal) (**A**,**C**–**F**). IgE-sensitized BMMCs were treated with Thal for 1 h and then stimulated with DNP-HSA for 15 min (**B**). The levels of phosphorylated or total protein of signaling molecules from whole cell lysates (WCL) or immunoprecipitates with anti-AMPKα antibody were determined by immunoblotting (**A**–**C**). The representative blots from three independent experiments were presented. Degranulation (**D**) and secretion of LTC_4_ (**E**) and PGD_2_ (**F**) were analyzed. Data indicate the means ± SEM (n = 3, ** *p* < 0.01 and *** *p* < 0.001 vs. Mock in each treatment; ^##^ *p* < 0.01 and ^###^ *p* < 0.001 vs. IgE/Ag alone in each group).

CRBN is a binding partner of AMPK and thalidomide [13,14]. Therefore, we assessed the involvement of CRBN in the effect of thalidomide on FcεRI-mediated mast cell activation by using CRBN siRNA. Genetic knockdown of CRBN substantially increased the levels of IgE/Ag-reduced phosphorylations of LKB1, AMPK, and ACC (Figure 3A and Figure S5). The IgE/Ag stimulation of BMMCs markedly increased the association of CRBN with AMPK, and this was attenuated by thalidomide. Thalidomide alone slightly decreased the basal level of interaction between AMPK and CRBN (Figure 3B). Under the same conditions, AMPK and CRBN were not precipitated with control IgG antibody (Figure S6). This indicates that CRBN negatively regulates the AMPK pathway in IgE/Ag-stimulated BMMCs by interacting with AMPK, and that thalidomide relieves suppression of the AMPK pathway mainly by interfering with the CRBN-AMPK interaction. CRBN siRNA consistently decreased the IgE/Ag-mediated activation of PLCγ1, ERK, JNK, and IKK, and enhanced the inhibitory effects of thalidomide on these FcεRI signaling components (Figure 3C and Figure S5). In addition, CRBN siRNA reduced the levels of p-AKT and p-p38, which are regulated by Syk but not by AMPK. Accordingly, IgE/Ag-stimulated degranulation and the production of LTC_4_ and PGD_2_ was significantly lower in CRBN siRNA-treated cells than in mock-treated cells; CRBN siRNA enhanced the inhibitory effects of thalidomide (Figure 3D–F). These results suggest that thalidomide inhibits IgE/Ag-induced BMMC activation by antagonizing the action of CRBN, which promotes FcεRI-mediated BMMC activation by suppressing the AMPK pathway.

### 3.4. Thalidomide Interferes with the Interaction of Syk with CRBN in IgE/Ag-Stimulated BMMCs

**Figure 4:**
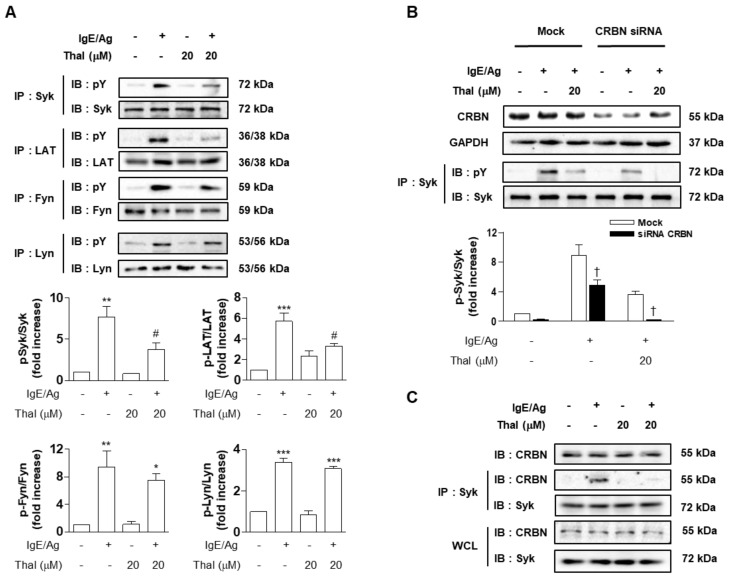
Thalidomide inhibits phosphorylation of Syk through interfering protein interactions of Syk with CRBN in IgE/Ag-stimulated BMMCs. BMMCs were sensitized with anti-DNP IgE and treated with thalidomide (Thal) for 1 h (**A**,**C**) or IgE-sensitized BMMCs were transfected with CRBN siRNA or non-specific (Mock) siRNA for 48 h and then treated with Thal (**B**), followed by stimulation with DNP-HSA (Ag) for 15 min. The immunoprecipitates or whole cell lysates (WCL) were subjected to immunoblot analysis with indicated antibodies. The immunoblot data are representative of three independent experiments. The bar graph presents the ratio of band intensity of phosphorylated signaling molecules to those of the total proteins (**A**,**B**). Data indicate the means ± SEM (n = 3, * *p* < 0.05, ** *p* < 0.01 and *** *p* < 0.001 vs. control; ^#^ *p* < 0.05 vs. IgE/Ag alone; † *p* < 0.05 vs. Mock in each treatment).

The above results suggest that thalidomide inhibits IgE/Ag-induced BMMC activation via both AMPK-dependent and -independent (Syk-dependent) signaling pathways. Therefore, we investigated the effect of thalidomide on the FcεRI-proximal tyrosine kinases Fyn, Lyn, and Syk in IgE/Ag-stimulated BMMCs. Thalidomide inhibited IgE/Ag-increased tyrosine phosphorylation (pY) of Syk and its target protein LAT; however, it did not affect the phosphorylation of Fyn and Lyn, which are upstream kinases of Syk (Figure 4A and Figure S6). CRBN siRNA substantially reduced IgE/Ag-increased Syk phosphorylation. The effect of thalidomide on Syk phosphorylation was much greater in CRBN siRNA-treated BMMCs than in mock-treated BMMCs (Figure 4B). IgE/Ag stimulation of BMMCs increased the association of CRBN with Syk, which was attenuated by thalidomide (Figure 4C and Figure S6). Therefore, it is plausible that the inhibitory effects of thalidomide on AMPK-independent signaling are mediated, at least in part, by the inhibition of Syk, which is positively regulated by CRBN.

### 3.5. Thalidomide Suppresses Syk Signaling by Activating SHP-1 in IgE/Ag-Stimulated BMMCs

**Figure 5:**
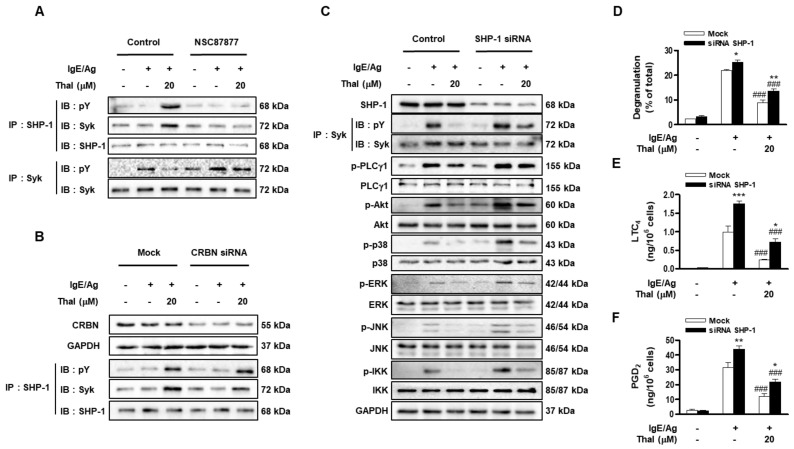
Thalidomide increases SHP-1 phosphorylation and protein interactions of SHP-1 with Syk in IgE/Ag-stimulated BMMCs. IgE-sensitized BMMCs were pretreated with 20 μM of NSC87877 (**A**), CRBN siRNA (**B**), or SHP-1 siRNA (**C**–**F**), and then stimulated with DNP-HSA (Ag) for 15 min in the presence or absence of thalidomide (Thal). The immunoprecipitates or whole cell lysates were subjected to immunoblot analysis and data are representative of three independent experiments (**A**–**C**). Degranulation (**D**) and the secretion of LTC_4_ (**E**) and PGD_2_ (**F**) were evaluated. Data indicate the means ± SEM (n = 3, * *p* < 0.05, ** *p* < 0.01, and *** *p* < 0.001 vs. Mock in each treatment; ^###^ *p* < 0.001 vs. IgE/Ag alone in each group).

In previous studies [33,34,35], we and others demonstrated that SHP-1, a protein tyrosine phosphatase, negatively regulates the phosphorylation of Syk in mast cells. Therefore, we assessed the effect of thalidomide on SHP-1 activation. As reported previously [34,35], the phosphorylation of SHP-1 and its interaction with Syk did not differ between basal and IgE/Ag-stimulated BMMCs (Figure 5A and Figure S7). However, thalidomide markedly enhanced the phosphorylation of SHP-1 and its association with Syk in unstimulated and FcεRI-stimulated cells (Figure 5A and Figure S8), and these effects were abolished by NSC87877, a SHP-1 inhibitor. Concurrently, NSC87877 reversed the antagonizing effect of thalidomide on Syk phosphorylation. CRBN knockdown had no effect on thalidomide-stimulated SHP-1 phosphorylation; however, it slightly decreased the interaction of SHP-1 with Syk which was induced by thalidomide (Figure 5B and Figure S7). This may be because CRBN siRNA decreased Syk phosphorylation. The role of SHP-1 in the inhibitory effects of thalidomide on Syk signaling was further examined by performing genetic knockdown of SHP-1. SHP-1 siRNA attenuated the inhibitory effects of thalidomide on Syk phosphorylation (Figure 5C and Figure S7). Consequently, SHP-1 siRNA increased the IgE/Ag-stimulated activation of Syk signaling pathways (PLCγ1, AKT, p38, ERK, JNK, and IKK) and attenuated the inhibitory effects of thalidomide on these signaling molecules. Consistent with the immunoblot analysis, SHP-1 siRNA largely abolished the inhibitory effects of thalidomide on IgE/Ag-induced degranulation and generation of eicosanoids (LTC_4_ and PGD_2_) (Figure 5D–F). These results indicate that thalidomide downregulates Syk signaling by activating SHP-1 in FcεRI-stimulated BMMCs.

### 3.6. Thalidomide Ameliorates the PCA Reaction in Mice

**Figure 6:**
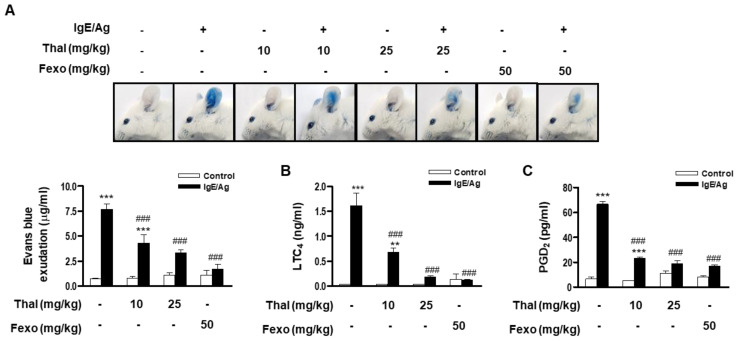
Thalidomide ameliorates IgE/Ag-mediated PCA reaction in mice. Mice were intradermally sensitized with IgE on the ears and were intravenously challenged with DNP-HSA (Ag) containing 1% evans blue. Thalidomide (Thal), fexofenadine (Fexo), or vehicle were given orally 1 h prior to DNP-HSA (Ag) challenge. The amount of dye extraction was measured as described in the Materials and Methods section and presented. Top panels show the representative photos of ears with dye extravasation (**A**). The levels of serum LTC_4_ (**B**) and PGD_2_ (**C**) were evaluated and data represent mean ± SEM (n = 6 mice per group; ** *p* < 0.01 and *** *p* < 0.001 vs. control in each treatment; ^###^ *p* < 0.001 vs. IgE/Ag alone).

The anti-allergic activity of thalidomide was further evaluated in an IgE-mediated PCA animal model. IgE-sensitized mice were orally administered thalidomide 1 h before being challenged with DNP-HSA. Then, the vascular permeability and serum levels of eicosanoids were evaluated. Fexofenadine, a selective histamine H1 receptor blocker, was used as a reference control. Thalidomide (10 and 25 mg/kg) significantly reduced IgE/Ag-increased dye extravasation and the serum levels of eicosanoids (LTC_4_ and PGD_2_) in a dose-dependent manner (Figure 6A–C). The inhibitory effects of 25 mg/kg thalidomide on the anaphylactic reaction were comparable with those of 50 mg/kg fexofenadine. These results demonstrate that thalidomide inhibits the FcεRI-mediated PCA reaction in vivo.

## 4. Discussion

Previous reports showing the anti-inflammatory function of thalidomide and inhibitory regulation of AMPK by CRBN indicated to us that thalidomide may have therapeutic effects on mast cell-mediated allergic diseases. In the present study, we showed that thalidomide inhibits FcεRI-mediated allergic reactions in mast cells in vitro and in an in vivo PCA model. Thalidomide decreased the association of CRBN with AMPK and thereby relieved the suppression of the AMPK pathway. In addition, thalidomide inhibited Syk signaling by activating SHP-1 and interfering with the interaction between Syk and CRBN. Thus, relief of the suppression of the AMPK pathway and activation of SHP-1 in parallel with disruption of the interaction between CRBN and Syk may underlie the suppression of FcεRI-mediated mast cell activation by thalidomide.

Several lines of evidence demonstrate that thalidomide inhibits CRBN, a binding partner of AMPK [14,36,37]. Consistently, AMPK knockdown attenuated sensitivity to thalidomide, whereas CRBN knockdown potentiated the effect of thalidomide on IgE/Ag-stimulated BMMCs. Therefore, thalidomide may elicit anti-allergic effects by positively and negatively regulating the actions of AMPK and CRBN, respectively, in mast cells. CRBN is a primary target protein of immunomodulatory drugs (IMiDs) such as thalidomide and its derivatives lenalidomide and pomalidoide, and mediates the teratogenic and antitumor effects of IMiDs via both ubiquitin-dependent and -independent pathways [38,39,40,41]. IMiDs are believed to compete with CRBN to bind to the CD147-MCT1 complex, which interferes with the activation of CD147-MCT1 by CRBN and, thereby, inhibits angiogenesis and cell proliferation [36]. Similarly, the present study demonstrated that thalidomide interferes with the interaction between CRBN and AMPK, which may be one of the mechanisms underlying the CRBN- and AMPK-mediated inhibitory effect of thalidomide on BMMC activation. By contrast, a recent study showed that thalidomide restores the weakened interaction between CRBN and AMPK under ischemic conditions and thereby inhibits AMPK activity and reduces neuronal cell death [42]. These observations suggest that the mode of regulation of CRBN and AMPK by thalidomide may be both cell type- and stimulus-specific. In addition, it has been reported that CRBN is a nucleocytoplasmic shuttling protein [43] that performs multiple functions in the cytosol and nucleus by interacting with different proteins, depending on the cell type and stimuli [14,38,44]. Notably, we previously reported that IgE/Ag-mediated stimulation of BMMCs leads to the nuclear translocation of LKB1/AMPK, thereby dampening their inhibitory effects on FcεRI signaling in the cytosol [7,8]. Our results demonstrate that thalidomide antagonizes the positive regulatory effect of CRBN on FcεRI signaling without affecting CRBN protein levels (Figure 3A). Therefore, it is possible that thalidomide attenuates FcεRI-mediated BMMC activation by altering the subcellular localization of CRBN and LKB1/AMPK, thereby disrupting the association between CRBN and AMPK.

CRBN is involved in multiple biological functions including cell metabolism, neuronal action, and autoimmune disorders [37], and mediates diverse effects of IMiDs by interacting with various protein partners. Although there are contrasting reports of the anti-inflammatory and pro-inflammatory roles of CRBN, our current results indicate that CRBN has a pro-inflammatory function in FcεRI-mediated mast cell activation. In addition to negatively regulating the AMPK pathway, CRBN increased FcεRI-stimulated BMMC activation partly by increasing Syk phosphorylation, which was substantially reduced by thalidomide. Although the precise mechanisms of action by which CRBN and thalidomide affect Syk phosphorylation have not been clearly elucidated, the thalidomide derivative lenalidomide reduces Syk phosphorylation by decreasing lymphocyte-specific-protein-kinase (Lck) phosphorylation in chronic lymphocytic leukemia cells [45]. Very recently, we also observed that the IgE/Ag stimulation of BMMCs increases Lck phosphorylation and that the inhibition of Lck by A770041, a selective inhibitor of Lck, significantly inhibits the IgE/Ag-induced degranulation and generation of LTC_4_ and PGD_2_. However, A770041 has no effect on Syk phosphorylation in IgE/Ag-stimulated BMMCs [46]. This suggests that the regulation of Syk phosphorylation by CRBN or thalidomide in IgE/Ag-stimulated BMMCs is mediated by signaling molecules other than Lck. Alternatively, it is possible that thalidomide causes degradation of the key signaling molecules involved in mast cell activation. It has been reported that immunomodulatory drugs (IMiDs), including thalidomide and its derivatives, inhibit or enhance the ubiquitination of CRBN-interacting proteins [47]. Among the genes that are downregulated, Ikaros family members IKZF1 (Ikaros) and IKZF3 (Aiolos) are the most affected by IMiD. Interestingly, Ikaros-/- mice exhibit a decreased number of intestinal mast cells [48]. Therefore, it is possible that thalidomide exerts anti-allergic activity in vivo via downregulating Ikaros protein and decreasing the mast cell development.

Upstream tyrosine kinases, such as Fyn and Lyn, and tyrosine phosphatases, such as SHP-1, regulate Syk signaling [33,34,49,50,51]. In the present study, thalidomide reduced the phosphorylation of Syk without affecting Fyn or Lyn, but significantly increased SHP-1 phosphorylation, suggesting that the effect of thalidomide on Syk phosphorylation is predominantly mediated by the SHP-1-dependent pathway. Indeed, thalidomide increased the activation of SHP-1 and the interaction between Syk and SHP-1. Moreover, SHP-1 knockdown markedly reduced the inhibitory effect of thalidomide on Syk phosphorylation. This suggests that thalidomide inhibits IgE/Ag-stimulated Syk phosphorylation by activating the SHP-1 pathway and interfering with the interaction between Syk and CRBN.

**Figure 7:**
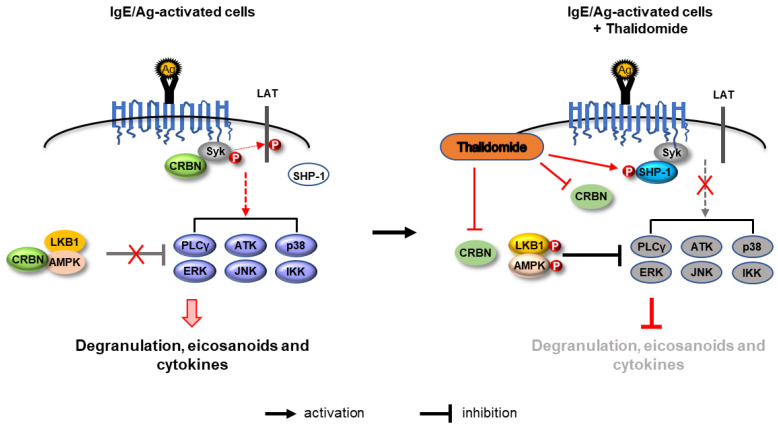
A possible mechanism by which thalidomide inhibits FcεRI-mediated mast cell activation. For details, please see text.

In summary, the binding of IgE/Ag to FcεRI promotes the interactions of CRBN with AMPK and Syk. These interactions facilitate mast cell activation by attenuating inhibitory signaling of LKB1 and AMPK and activating stimulatory signaling of the Syk pathway. Thalidomide attenuates FcεRI-stimulated mast cell activation by relieving the suppression of the LKB1/AMPK pathway and activating SHP-1 signaling, while simultaneously interfering with the interaction between CRBN and Syk. These results are summarized in Figure 7. Our findings provide evidence that thalidomide suppresses IgE/Ag-stimulated mast cell activation by upregulating the LKB1/AMPK and SHP-1 signaling modules and downregulating the action of CRBN. This indicates that thalidomide may be a promising therapeutic agent for the treatment of mast cell-mediated allergic diseases.

## Data Availability

Not applicable.

## Supplementary Materials

The following supporting information can be downloaded at: https://www.mdpi.com/article/10.3390/cells12030469/s1, Figure S1: The effects of thalidomide on viability of mast cells; Figure S2: Densitometric analysis of the effects of thalidomide on the phosphorylation levels of signaling molecules in BMMCs; Figure S3. Inhibitory effects of thalidomide on IgE/Ag-stimulated degranulation and eicosanoid production in RBL2H3 cells; Figure S4: Densitometric analysis of the effects of AMPKα knockdown on the phosphorylation levels of signaling molecules in BMMCs; Figure S5: Densitometric analysis of the effects of CRBN knockdown on the phosphorylation levels of signaling molecules in BMMCs; Figure S6. Interaction between AMPK, Syk, SHP-1, LAT, Lyn, Fyn, or CRBN and normal IgG. Figure S7: Densitometric analysis of the effects of SHP-1 inhibition on the levels of phosphorylation or protein interaction of signaling molecules in BMMCs: Figure S8. The effects of thalidomide on SHP-1 phosphorylation and interaction between of SHP-1 and Syk.
