# Supplementary material for: Thalidomide Attenuates Mast Cell Activation by Upregulating SHP-1 Signaling and Interfering with the Action of CRBN
Source: Cells. 2023 Feb 1;12(3):469. doi: 10.3390/cells12030469 (PMC9914299; doi:10.3390/cells12030469)
Supplement: Supplementary file 1 [file cells-12-00469-s001.zip › cells-2126548-Supplementary materials.pdf]

Supplementary Materials for:

Thalidomide attenuates mast cell activation by upregulating SHP-1 signaling and interfering with the action of CRBN

Hyeun Wook Chang [1], Kyeong Hwa Sim [2] and Youn Ju Lee [2]*

[1] College of Pharmacy, Yeungnam University, Gyeongsan, Gyeongbuk 38541, Republic of Korea
[2] Department of Pharmacology, School of Medicine, Daegu Catholic University, Daegu, 42472, Republic of Korea
* Correspondence: whrytn4337@cu.ac

Figure S1.

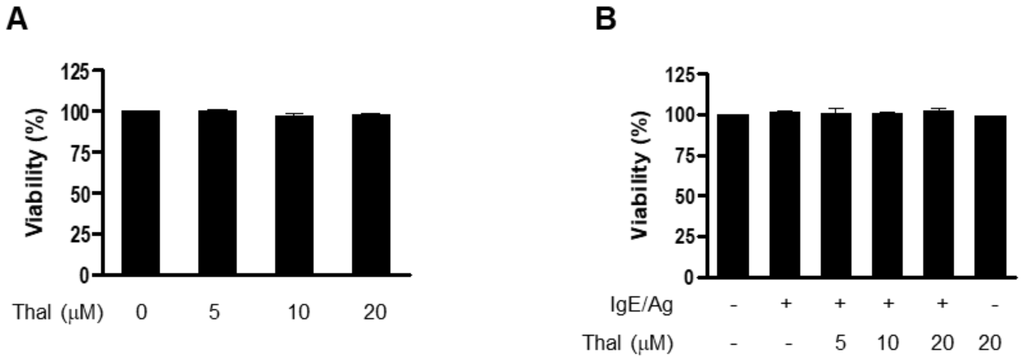

Figure S1. The effects of thalidomide on viability of mast cells. BMMCs were treated with different concentrations (0, 5, 10, and 20 μM) of thalidomide for 10 h (A) or IgE-sensitized BMMCs were treated with different concentrations (0, 5, 10, and 20 μM) of thalidomide for 1 h and then stimulated with Ag (DNP-HSA) for 15 min. (B) Cell viability was measured by MTT assay using tetrazolium salt 3-[4,5-dimethylthiazol-2-yl]-2,5- diphenyltetrazolium bromide (MTT).

Figure S2.

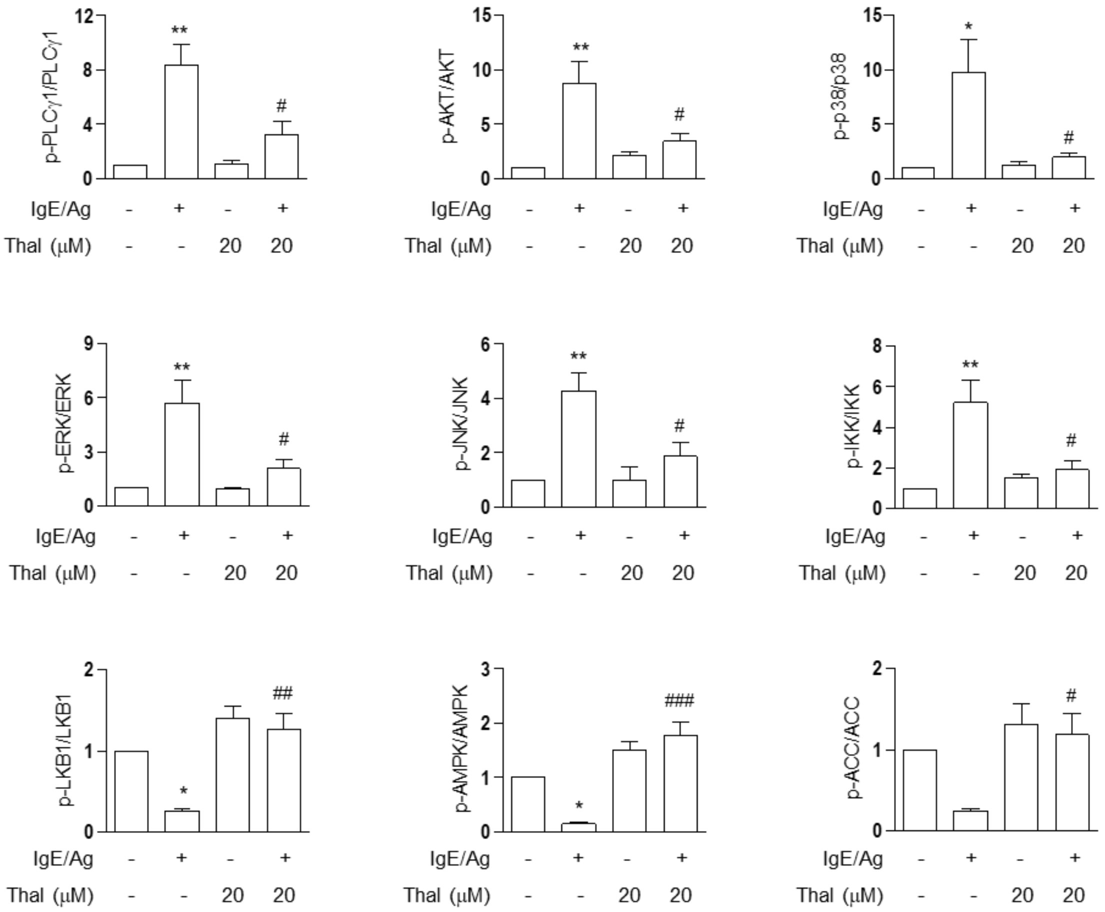

Figure S2. Densitometric analysis of the effects of thalidomide on the phosphorylation levels of signaling molecules in BMMCs. The ratios of the band intensities of phosphorylated signaling molecules to those of the total proteins (Fig. 1J) were determined using the Chemi-Doc XRS imaging system (Bio-Rad, Hercules, CA). Data from three independent experiments are expressed as fold increases in the bar graphs ($^{*}P < 0.05$ and $^{**}P < 0.01$ *vs.* control; $^{\#}P < 0.05$, $^{\#\#}P < 0.01$ and $^{\#\#\#}P < 0.001$ *vs.* IgE/Ag alone).

Figure S3.

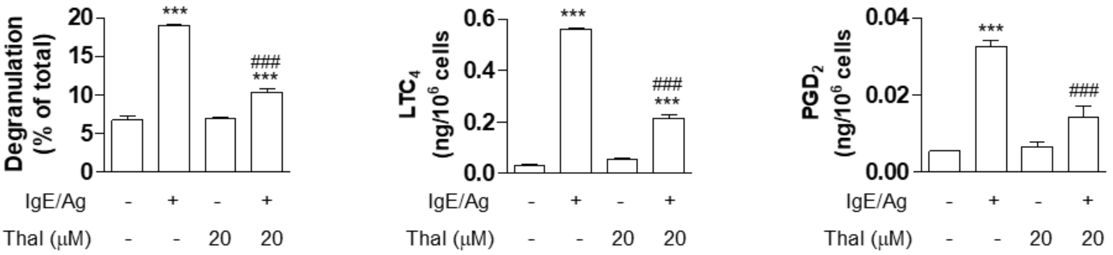

Figure S3. Inhibitory effects of thalidomide on IgE/Ag-stimulated degranulation and eicosanoid production in RBL2H3 cells. IgE-sensitized RBL2H3 cells were treated with 20 μM of thalidomide for 1 h and then stimulated with Ag (DNP-HSA). Degranulation and secretion of LTC_4 and PGD_2 were evaluated. ($^{***}P < 0.001$ *vs.* control; $^{\#\#\#}P < 0.001$ *vs.* IgE/Ag alone).

Figure S4.

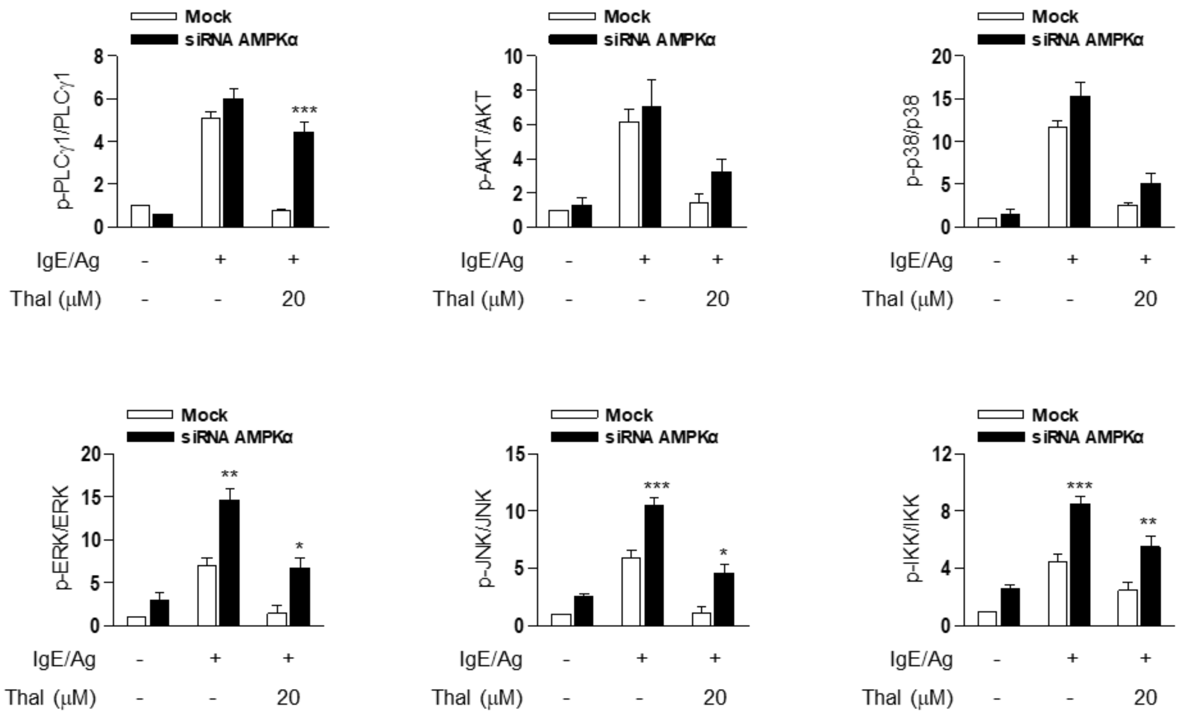

Figure S4. Densitometric analysis of the effects of AMPKα knockdown on the phosphorylation levels of signaling molecules in BMMCs. The ratios of the band intensities of phosphorylated signaling molecules to those of the total proteins (Fig. 2B) were determined using the Chemi-Doc XRS imaging system (Bio-Rad, Hercules, CA). Data from three independent experiments are expressed as fold increases in the bar graphs ($^{*}P < 0.05$, $^{**}P < 0.01$, and $^{***}P < 0.001$ *vs.* Mock in each treatment).

Figure S5.

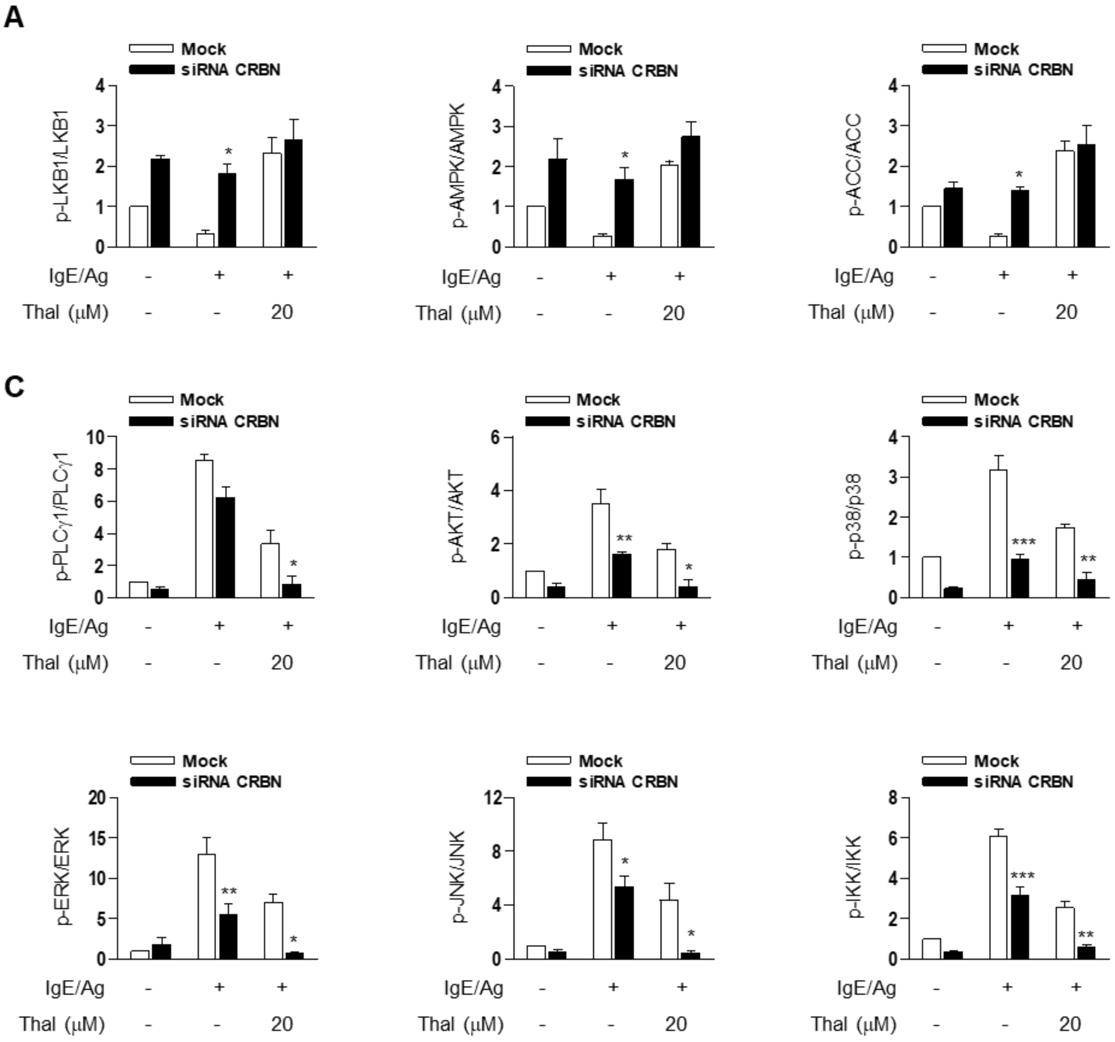

Figure S5. Densitometric analysis of the effects of CRBN knockdown on the phosphorylation levels of signaling molecules in BMMCs. The ratios of the band intensities of phosphorylated signaling molecules to those of the total proteins (Fig. 3A, 3C) were determined using the Chemi-Doc XRS imaging system (Bio-Rad, Hercules, CA). Data from three independent experiments are expressed as fold increases in the bar graphs ($^{*}P < 0.05$, $^{**}P < 0.01$, and $^{***}P < 0.001$ *vs.* Mock in each treatment).

Figure S6.

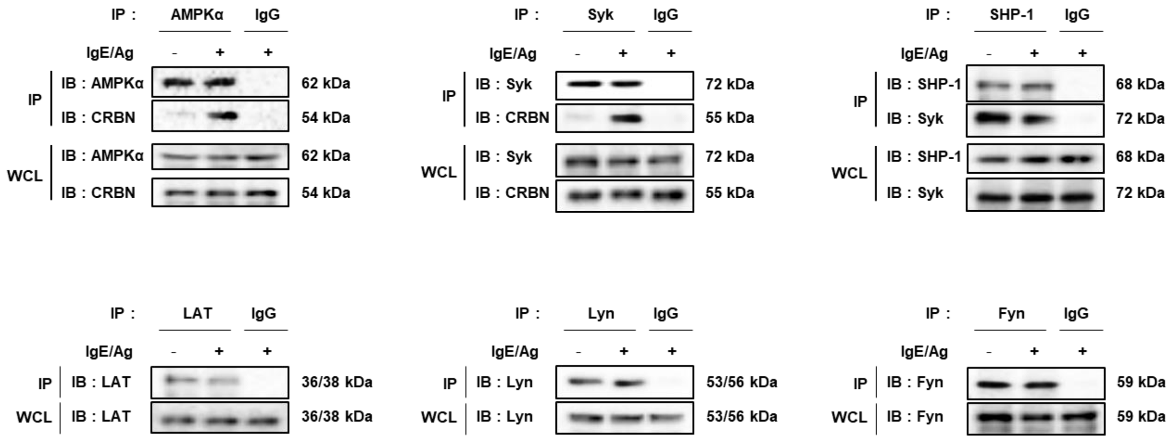

Figure S6. Interaction between AMPK, Syk, SHP-1, LAT, Lyn, Fyn, or CRBN and normal IgG. BMMCs were sensitized with IgE and then stimulated with or without DNP-HSA for 15 min. Cell lysates were subjected to immunoprecipitation using anti-AMPK, -Syk, -SHP-1, -LAT, -Lyn, -Fyn antibodies or IgG followed by immunoblotting using specific antibodies. Normal IgG was used as a negative control. Whole cell lysates (WCL) were subjected to immunoblotting with specific antibodies and used as input controls.

Figure S7.

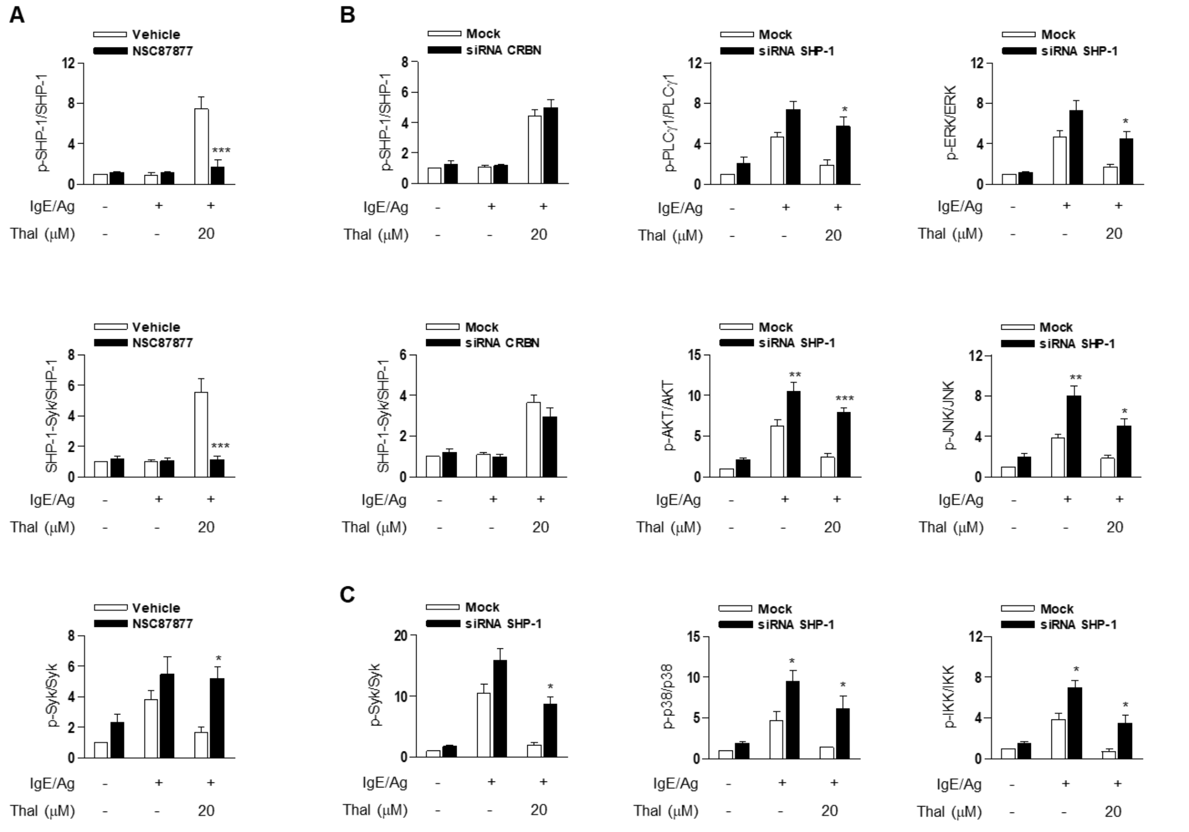

Figure S7. Densitometric analysis of the effects of SHP-1 inhibition on the levels of phosphorylation or protein interaction of signaling molecules in BMMCs. The ratios of the band intensities of phosphorylated or associated signaling molecules to those of the total proteins (Fig. 5A-C) were determined using the Chemi-Doc XRS imaging system (Bio-Rad, Hercules, CA). Data from three independent experiments are expressed as fold increases in the bar graphs ($^{*}P < 0.05$, $^{**}P < 0.01$, and $^{***}P < 0.001$ *vs.* Mock or vehicle in each treatment).

Figure S8.

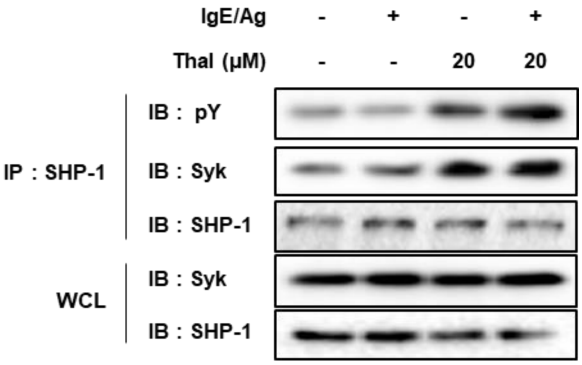

Figure S8. The effects of thalidomide on SHP-1 phosphorylation and interaction between of SHP-1 and Syk. IgE-sensitized BMMCs were treated with thalidomide (Thal) for 1 h and then stimulated with or without DNP-HSA for 15 min. Cell lysates were subjected to immunoprecipitation using anti-SHP-1 followed by immunoblotting using specific antibodies. Whole cell lysates (WCL) were subjected to immunoblotting with specific antibodies and used as input control.
